# Diagnostic Concordance of Cytology and Histology in Samples Obtained via Endoscopic Ultrasound-Guided Fine-Needle Biopsy (EUS-FNB)

**DOI:** 10.7759/cureus.15596

**Published:** 2021-06-11

**Authors:** Tara Keihanian, Liege Diaz, Liza Plafsky, Uday Shergill, Jinendra Satiya, Rtika Abraham, Monica Garcia-Buitrago, James H Tabibian, Mohit Girotra

**Affiliations:** 1 Gastroenterology, University of Miami Miller School of Medicine, Miami, USA; 2 Gastroenterology and Therapeutic Endoscopy, Swedish Medical Center, Seattle, USA; 3 Gastroenterology and Hepatology, Jackson Memorial Hospital, Miami, USA; 4 Internal Medicine, Jackson Memorial Hospital, Miami, USA; 5 Pathology, Jackson Memorial Hospital, Miami, USA; 6 Gastroenterology and Hepatology, Beth Israel Deaconess Medical Center, Harvard Medical School, Boston, USA; 7 Endocrinology, Diabetes and Metabolism, University of Miami Miller School of Medicine, Miami, USA; 8 Pathology, University of Miami, Miami, USA; 9 Gastroenterology, David Geffen School of Medicine at University of California Los Angeles, Los Angeles, USA; 10 Gastroenterology, Olive View-University of California Los Angeles Medical Center, Sylmar, USA; 11 Gastroenterology and Hepatology; Advanced Endoscopy, University of Miami Miller School of Medicine, Miami, USA

**Keywords:** endoscopic ultrasound (eus), fine-needle aspiration, fna cytology, eus fna

## Abstract

Introduction

Endoscopic ultrasound (EUS)-guided fine-needle aspiration and biopsy (FNA/FNB) to obtain cytological aspirates and histological core samples, respectively, are the standard of care for diagnosing lesions in/adjacent to the upper/lower gastrointestinal tract. Due to the lack of standardization of tissue processing, it is unclear whether core samples should be sent only for histology (formalin) or cytology (CytoLyt), or both. The aim of this study was to investigate the diagnostic concordance rates between cytology and histology on EUS-FNB core samples.

Methods

A total of 227 patients underwent EUS-FNB between October-2017 and February-2019 by a single therapeutic endoscopist; 44 core-tissue samples (41 patients) were placed alternately in CytoLyt (cytology) and formalin (histology), with equal passes into each, to best achieve a proportionate sample amount. The patient's demographics, medical history, pertinent imaging, EUS indication/findings were reviewed. Main outcomes included concordance rates between cytology-histology and diagnostic accuracy for malignancy.

Results

Cytology and histology were discordant in five cases (11.5%); four with negative cytology but a definite diagnosis of malignancy achieved with histology. One case was suspected as neoplasm on cytology but further characterized as benign on histology. Cytology failed to sub-characterize an additional four mass-like pancreatic benign entities, due to inadequate tissue architecture assessment in the CytoLyt sample. Sensitivity, specificity, positive predictive value (PPV) and negative predictive value (NPV) of cytology for diagnosis of malignancy were 87.88% (95%CI: 71.8-96.6), 90.91% (95%CI: 58.7-99.7), 96.67% (95%CI: 81.6-99.4), and 71.43% (95%CI: 49.4-86.4).

Discussion

We observed 11.5% diagnostic discordance between cytology and histology on EUS-FNB core samples, with histology being superior. Future multicenter prospective randomized studies are needed to establish an accurate and cost-effective diagnostic process.

## Introduction

The advent of endoscopic ultrasound (EUS) has vastly improved access to pancreatic and other gastrointestinal (GI) lesions, leading to increased diagnostic capabilities. EUS-guided tissue acquisition (EUS-TA) using fine-needle aspiration or biopsy (FNA/FNB) is now the accepted standard of care for the diagnosis of solid masses/lesions in proximity to the GI tract [1-2]. FNA helps in the acquisition of individual cells for cytology while FNB provides a histological core specimen useful for immunohistochemistry and the evaluation of tissue architectural changes [3].

FNA cytology has been traditionally favored for pancreatic lesions, for their additional advantage for next-generation sequencing (NGS), as cytological samples are believed to contain a higher concentration of pure tumor cells, and the alcohol-based fixation of the samples improves nucleic acid preservation [4]. However, FNB appears to be superior for lesions other than solid pancreatic masses and peripancreatic lymph nodes, and both 22 and 25-gauge core needles can provide a high diagnostic yield with equivalent accuracy on histology [5]. Recent advances have greatly improved EUS-TA with fewer needle passages required to establish a diagnosis, especially at centers where rapid on-site evaluation (ROSE) is available [6]. However, ROSE is not offered at a majority of centers worldwide, and therefore, the practice of handling procured specimens is individualized based on the operator’s preferences and expertise. There is no universally accepted technique for specimen collection and processing, which often affects diagnostic accuracy. Furthermore, there is no consensus on how ROSE should be performed, with current practice possibilities including reviewing smears in the procedure area, transferring slides to the pathologists’ office, or evaluating slides via telepathology [4,7].

In addition to these inconsistencies, there is no standardization if the diagnostic sample should be sent for cytology (CytoLyt), histology (formalin), or both. After EUS-TA, the sample can be processed using two different techniques: in cytological preparation, the cell aspirate is smeared on a slide, which is placed in a container with a cytology fixative (i.e. CytoLyt for liquid-based cytology (LBC), Sacommanno for cytospin); while in histological preparation, the aspirated cells and small core fragments are fixed in 10% formalin for routine histologic assessment. In our practice, we encountered a few cases where although cytology was negative, when a repeat sample was sent in formalin for histology, a diagnosis of malignancy was achieved. The present study was hence undertaken to evaluate if the diagnostic yield of cytology is less or equal to histology when the sample is obtained with the same EUS-FNB needle, in order to ascertain the best practice and make the diagnostic process more accurate and universal.

## Materials and methods

The present study was conceptualized as an investigator-initiated, prospectively maintained database, which was later retrospectively analyzed. The Institutional Review Board at the University of Miami Hospitals and Clinics (UMHC) approved the study (IRB#20181076).

Patient selection

**Figure 1:**
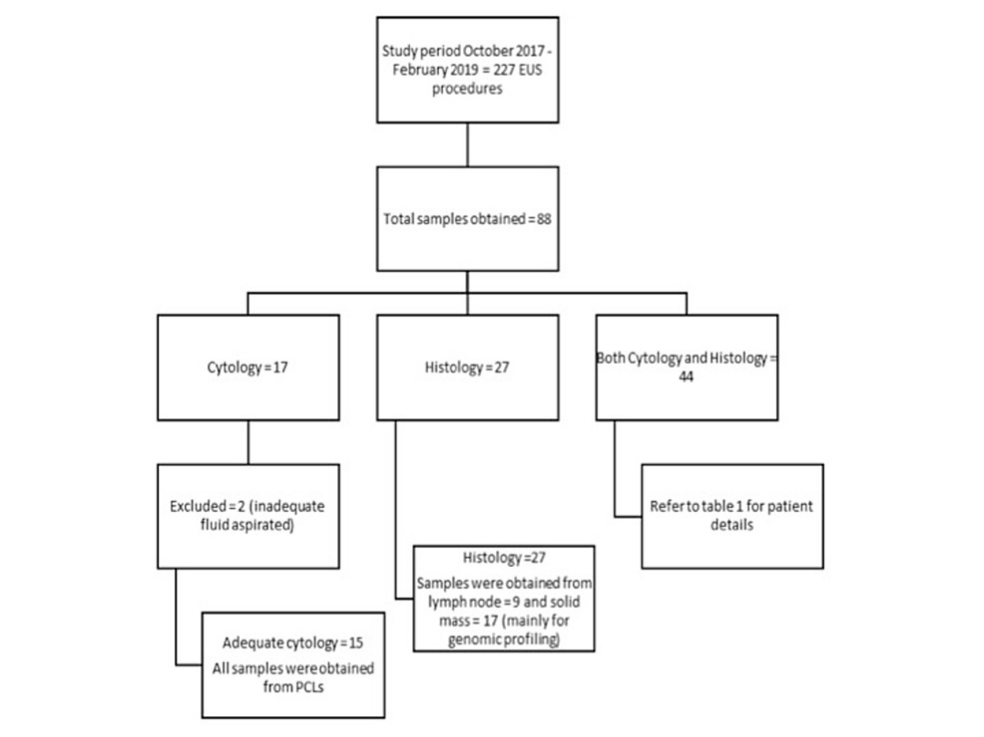
Patient recruitment design flowchart

Between October 2017 and February 2019, a total of 227 patients underwent EUS by a single therapeutic endoscopist (M.G.), at a large tertiary referral center, for abnormal imaging findings and/or clinical symptoms, out of which 88 patients required EUS-TA. Forty-four (44) specimens, obtained from 41 patients, were ascertained as adequate/appropriate to be sent for both histology and cytology analysis. In an additional 17 patients (with pancreatic cystic lesions, PCLs), only fluid cytology was sent, and in the remaining 27 patients, the specimen was sent only for histology, as requested by oncology for specific bulk ribonucleic acid (RNA) sequencing/genomic profiling of known tumors (Figure 1).

Study design and variables collected

In 41 patients, core tissue obtained from 44 lesions, using a core FNB needle, was alternated between CytoLyt (for cytology) and formalin (for histology), with a goal to achieve an equal amount of specimen in both, either through an equal number of passes assigned to each or through visual inspection of obtained tissue material floating in the solution bottles. The FNB technique remained consistent throughout these 44 procedures, with the passage of a 22-gauge FNB needle (one case had the additional use of a 25-gauge FNB needle) into the suspected lesion and then moved back-and-forth in a fanning fashion while withdrawing the needle plunger to create negative pressure to suction core-tissue into the needle. The sample collected for cytology was first used to prepare two conventional smears (CS) on a glass slide, and then the visible tissue in the core needle was placed in CytoLyt (Hologic Inc., Marlborough, MA) for LBC while the sample collected for histology was placed in 10% formalin directly. Five GI pathologists evaluated the EUS-FNB samples obtained in formalin while the CytoLyt specimens were assessed by certified cytopathologists. All discordant cases were individually reviewed by a GI pathology fellow (U.S.) with an expert GI pathologist, with additional training and experience in cytopathology (M.G.B).

The data collected included patients’ demographics, medical history, imaging findings [prior computed tomography (CT)/magnetic resonance imaging (MRI)], EUS indications/findings, and cytology/histology results.

Data analysis

The Statistical Package for the Social Sciences (SPSS) version22 (IBM Corp, USA) was utilized to perform statistical analysis. Frequencies and percentages were calculated for categorical variables. Continuous variables were summarized with mean ± standard deviation.

## Results

Study design

Out of 88 EUS-TA procedures, 44 samples (in 41 patients) were analyzed for both cytology and histology while the remaining samples were sent for only cytology (n=17) or only histology (n=27), depending on the nature of the lesion or indication, as explained above. The 27 histology-only (formalin) samples were obtained using 22-gauge FNB needles in all cases, except in one where a 25-gauge FNB needle was used. The final diagnosis was achieved in all 27 cases, which were analyzed histologically. On the other hand, out of 17 cytology-only (CytoLyt) samples, the amount of aspirated fluid was inadequate in two cases, and final diagnosis could not be achieved in two out of 15 cases (all PCLs) and required further investigation.

Patient and lesion characteristics

The focus of our analysis was the 41 patients in whom 44 procured tissue samples were sent for both cytology and histology analysis (see Table 1), which included 27 solid pancreatic solid masses, five stomach lesions, four liver lesions, three lymph nodes, and one each of ampullary, duodenal, esophageal, peri-gastric, and surgical bed lesions. Three of these 41 patients had two lesions each, the first with two pancreatic masses, the second with a pancreatic mass and lymph node, and the third with a pancreatic mass and liver lesion (see the last three rows of Table 1). These lesions were biopsied using different core needles and hence included as distinct lesions in the data analysis. The standard needle of choice was a 22-gauge core FNB needle in all 44 cases, and, additionally, in one case, two initial passes were made with a 25-gauge FNB needle, given the difficult location of the ampullary lesion (see Table 1). The mean age of patients was 63.32±10.06 years, and 61% were males (25/41). The mean size of these 44 biopsied lesions was 34±17.58 mm, which included a few large gastric masses. The mean size of pancreatic lesions was 32.26±9.41 mm. No EUS-related major complications (perforation, bleeding, infection/fever) were noted in any of the procedures.

**Table 1:** Demographic, procedural, and EUS-FNB specimen characteristics of 41 patients with 44 target lesions * These three patients had multiple lesions, which were separately sampled using separate needles. ! These six patients had concordant cytology and histology, and both were negative for malignancy. $ These two patients had non-diagnostic cytology but abnormal cells were seen. EUS: endoscopic ultrasound; FNB: fine-needle biopsy

Age/ Gender	Target site	Sample length	Needle gauge	Needle passes	Cytology diagnosis	Histology diagnosis	Diagnostic Advantage
60/M	Lymph node (Peri-esophageal)	17 x 6.2 mm	22	5	Nondiagnostic -lymph node tissue	Follicular lymphoma	Histology
65/M	Pancreatic body-tail lesion	27 x 27 mm	22	4	Non- diagnostic	Inflammation and increased IgG4 compatible with IgG4 Pancreatitis	Histology
73/F	Gastric lesion	51 x 27 mm	22	6	Nondiagnostic -negative for malignancy	Gastrointestinal stromal tumor	Histology
71/M	Pancreatic body lesion	38 x 38 mm	22	6	Nondiagnostic - negative for malignancy	Chronic pancreatitis	Histology
65/M	Pancreatic body lesion	33 x 32 mm	22	5	Nondiagnostic - negative for malignancy	Splenule	Histology
82/M	Pancreatic tail lesion	20 x 18 mm	22	6	Nondiagnostic - negative for carcinoma	Lymphoid and connective tissue with non-necrotizing epithelioid granulomas	Histology
49/F	Peri-gastric mass	89 x 55 mm	22	5	Spindle cell neoplasm	Venous hemangioma	Histology
49/M^$^	Pancreatic head lesion	32 x 32 mm	22	6	Scattered abnormal ductal cells	Adenocarcinoma	Histology
67/M^$^	Pancreatic head/neck lesion	31 x 27 mm	22	6	Rare abnormal cells of epithelial origin	Adenocarcinoma	Histology
53/M	Pancreatic neck lesion	22 x 22 mm	22	4	Neuroendocrine neoplasm	Neuroendocrine tumor, WHO grade I	Equivalent
60/M	Pancreatic head lesion	29.8 x 30.1 mm	22	5	Adenocarcinoma	Adenocarcinoma	Equivalent
61/M	Liver lesion	15.4 x 13 mm	22	6	Squamous cell carcinoma	Squamous cell carcinoma	Equivalent
63/M	Pancreatic body/tail lesion	51 x 33 mm	22	6	Adenocarcinoma	Adenocarcinoma	Equivalent
82/F	Pancreatic head lesion	32 x 33 mm	22	5	Adenocarcinoma	Adenocarcinoma	Equivalent
73/F	Liver lesion	69 x 68 mm	22	6	Squamous cell carcinoma	Squamous cell carcinoma	Equivalent
52/M	Pancreatic head lesion	37 x 25 mm	22	5	Poorly differentiated carcinoma	Poorly differentiated carcinoma	Equivalent
76/F	Pancreatic head lesion	25.8 x 23.2 mm	22	6	Adenocarcinoma	Adenocarcinoma	Equivalent
67/M	Pancreatic tail lesion	45 x 40 mm	22	5	Adenocarcinoma	Adenocarcinoma	Equivalent
57/F	Pancreatic neck lesion	42 x 25 mm	22	5	Adenocarcinoma	Adenocarcinoma	Equivalent
70/M	Pancreatic neck/body lesion	55 x 55 mm	22	6	Squamous cell carcinoma	Squamous cell carcinoma	Equivalent
67/F	Gastric lesion	16 x 9 mm	22	4	Gastrointestinal stromal tumor	Gastrointestinal stromal tumor	Equivalent
50/F	Lymph node	67 x 50 mm	22	4	Plasma cell myeloma	Plasma cell myeloma	Equivalent
73/M	Pancreatic tail lesion	10 x 8 mm	22	5	Neuroendocrine tumor	Neuroendocrine tumor	Equivalent
71/F	Duodenal lesion	35 x 32 mm	22	6	Gastrointestinal stromal tumor	Gastrointestinal stromal tumor	Equivalent
59/F	Ampullary lesion	30 x 26 mm	25/22	2/4	Adenocarcinoma	Adenocarcinoma	Equivalent
62/M	Gastric lesion	83.9 x 53 mm	22	6	Gastrointestinal stromal tumor	Gastrointestinal stromal tumor	Equivalent
69/M	Pancreatic head lesion	29.6 x 26.4 mm	22	6	Poorly differentiated carcinoma w	Poorly differentiated carcinoma	Equivalent
60/F	Pancreatic body lesion	38 x 31 mm	22	6	Adenocarcinoma	Adenocarcinoma	Equivalent
62/F	Pancreatic tail lesion	26.7 x 25.7 mm	22	6	Neuroendocrine tumor	Neuroendocrine tumor	Equivalent
50/M	Gastric lesion	22.8 x 21.7 mm	22	4	Adenocarcinoma	Adenocarcinoma	Equivalent
81/M	Pancreatic head lesion	37.5 x 30.4 mm	22	5	Adenocarcinoma	Adenocarcinoma	Equivalent
78/F	Pancreatic neck lesion	35 x 37 mm	22	6	Adenocarcinoma	Adenocarcinoma	Equivalent
69/F^!^	Pancreatic body lesion	21.4 x 11.8 mm	22	6	Serous cyst neoplasm	Serous cystic neoplasm with atypical cells	Equivalent
35/M^!^	Esophageal lesion	38 x 18 mm	22	7	Leiomyoma	Leiomyoma	Equivalent
67/F^!^	Liver lesion	12.2 x 12.9 mm	22	3	Negative for malignancy	Negative for malignancy	Equivalent
60/M^!^	Pancreatic body lesion	24 x 24 mm	22	5	Intra-pancreatic spleen	Intra-pancreatic spleen	Equivalent
57/M^!^	Gastric lesion	22 x 23 mm	22	6	Negative for malignancy	Gastric mucosa	Equivalent
51/M^!^	Pancreatic head lesion	33 x 36 mm	22	6	Negative for malignancy	Negative for malignancy	Equivalent
62/M*	Pancreatic body lesion, Liver lesion	35 x 33 mm, 16 x 21 mm	22, 22	5, 5	Adenocarcinoma, Adenocarcinoma	Adenocarcinoma, Adenocarcinoma	Equivalent, Equivalent
64/M*	Prior pancreatic resection bed lesion, Lymph node	13.6 x 13.1 mm, 10 x 6 mm	22, 22	4, 4	Adenocarcinoma, Adenocarcinoma	Adenocarcinoma, Adenocarcinoma	Equivalent, Equivalent
54/F*	Pancreatic mid-body lesion, Pancreatic head lesion	28 x 25 mm, 26 x 26 mm	22, 22	6, 4	Adenocarcinoma, Adenocarcinoma	Adenocarcinoma, Adenocarcinoma	Equivalent, Equivalent

EUS-FNB cytology and histology comparison

**Figure 2:**
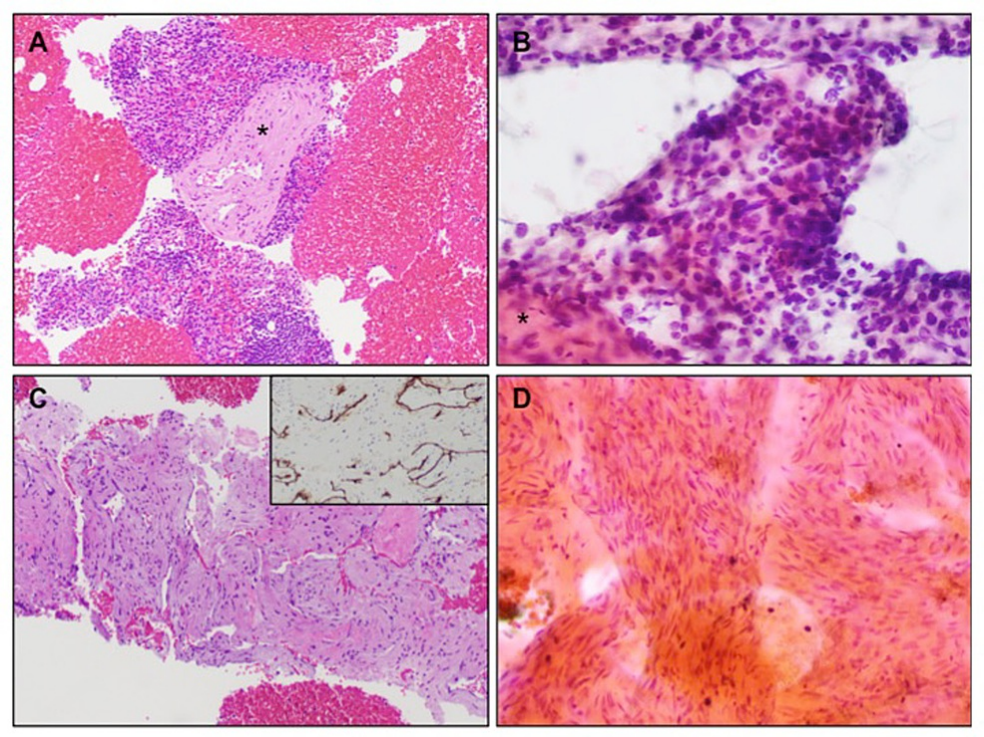
Histology and cytology of discordant cases Histology of a 17 x 11 mm hypoechoic peri-pancreatic lymph node (A) showing benign lymphoid tissue with well-delineated trabeculae (asterisk), consistent with splenic tissue/splenule. The concurrent cytology (B) showed lymphoid tissue and scant, ill-defined fibrous tissue (asterisk), which was histologically not suggestive of splenic trabeculae, and the aspirate was called benign lymphoid tissue. Histology of a peri-gastric mass (C) showing abnormal venous structures with prominent spindled smooth-muscle walls and luminal CD34 positive benign endothelial cells (inset), suggestive of venous hemangioma. Concurrent cytology (D) showed a proliferation of bland spindle cells raising concern for spindle-cell neoplasm.

Out of these 44 samples sent for both cytology and histology, 35 samples showed concordance between cytology and histology results (tumor in cytology/tumor in histology; no tumor in cytology/no tumor in histology). In nine samples (9/44 = 20.45%), histology provided a conclusive diagnosis. The source lesions in these nine cases included six pancreatic masses, one gastric subepithelial lesion, one peri-gastric lesion, and one lymph node along the anterior wall of the esophagus. In four cases with mass-forming pancreatic lesions, cytology confirmed the absence of malignancy/tumor, failed to provide a specific diagnosis, but was adequately diagnosed with histology specimen as immunoglobulin G4 (IgG4) autoimmune pancreatitis (AIP), splenule, chronic pancreatitis, and non-necrotizing epithelioid granulomata. These entities are less often expected to be conclusively diagnosed with cytology alone, which fails to provide the architectural details needed for their diagnosis and hence are diagnosed with histology specimen. Excluding these four, the remaining five lesions had true discordance between cytology and histology. In four of these cases [obtained from pancreatic lesions (n=2), gastric mass (n=1), and lymph node (n=1)], histology confirmed the diagnosis of malignancy while cytology was negative. In one peri-gastric mass, the cytology raised suspicion for spindle-cell neoplasm, however, histology clarified it to be a venous hemangioma with abnormal venous structures and prominent spindled smooth muscle walls (see Figure 2 and Table 2).

**Table 2:** Characteristics of patients with discordant results (N=9) IgG4: immunoglobulin G4

Characteristics	Data
Gender (Male: Female)	7:2
Age	64.5±10.15
Lesion size	37.5±20.45 mm
Location of lesions: Lymph node along the anterior wall of the esophagus, Gastric sub-epithelial lesion, Pancreas, Peri-gastric	1, 1, 6, 1
Final Diagnosis on histology: Lymph node (n=1), Gastric sub-epithelial lesion (n=1), Pancreas mass-like lesions (n=6), Peri-gastric lesion (n=1)	Follicular lymphoma, Gastrointestinal stromal tumor (GIST), Adenocarcinoma (x 2), IgG4 pancreatitis, Chronic pancreatitis, Splenule, Lymphoid and connective tissue with non-necrotizing epithelioid granulomas, Venous hemangioma

Of the samples analyzed with both cytology and histology, 29 were concordantly positive for malignancy, and an additional four samples with non-malignant cytology were proven to be malignant on histology. One peri-gastric lesion with neoplastic cytology was further characterized and confirmed to be non-malignant (venous hemangioma) on histology, which was later established on surgical pathology also. The remaining 10 samples (including the four mass-forming pancreatic lesions) were found to be concordantly negative for malignancy with both cytology and histology, and this was confirmed with serial secondary imaging (CT/MRI) as well. These data suggest a modest sensitivity of 87.88% (95%CI: 71.8-96.6) and accuracy of 88.6% (95%CI: 75.4-96.2), with a high specificity of 90.91% (95%CI: 58.7-99.7) and a positive predictive value (PPV) of 96.67% (95%CI: 81.6-99.4) but a low negative predictive value (NPV) of 71.43% (95%CI: 49.4-86.4) for cytology for the diagnosis of malignancy. Among the four non-malignant cytology cases, which were later proven malignant with histology, two cytology cases had atypical cells suspicious for carcinoma, and if those are taken into account, the sensitivity increases to 93.9% (95%CI: 79.9-99.2), NPV increases to 83.3% (95%CI: 56.3-95.1), and accuracy increases to 93.1% (95%CI: 81.3-98.5) (see Table 3). Among different neoplastic cases, compared to histology as the gold standard, the cytology detection rate of malignant epithelial neoplasms (adenocarcinoma, squamous cell carcinoma, poorly differentiated carcinoma) was 91.6% (22/24 cases), for mesenchymal neoplasms (GIST) was 75% (3/4 cases), for lymphoproliferative disorders (multiple myeloma and lymphoma) was 50% (1/2 cases), for benign epithelial neoplasms (serous cystadenoma) was 0% (0/1 cases), and for neuroendocrine tumors was 100% (3/3 cases).

**Table 3:** Performance of cytology for diagnosis of malignancy PPV: positive predictive value; NPV: negative predictive value; TP: true positives; FP: false positives; TN: true negatives; FN: false negatives

	Malignancy Positive	Malignancy Negative	
Cytology Positive	TP = 29	FP = 1	PPV = 96.67% (95% CI: 81.6-99.4)
Cytology Negative	FN = 4	TN = 10	NPV = 71.43% (95% CI: 49.4-86.4)
	Sensitivity = 87.88% (95% CI: 71.8-96.6)	Specificity = 90.91% (95% CI: 58.7-99.7)	Accuracy = 88.6% (95% CI: 75.4-96.2)
	Malignancy Positive	Malignancy Negative	
Cytology Positive + Atypical Cells	TP = 31	FP = 1	PPV = 96.8%(95% CI: 82.6-99.5)
Cytology Negative	FN = 2	TN = 10	NPV = 83.3% (95% CI: 56.3-95.1))
	Sensitivity = 93.9% (95% CI: 79.9-99.2)	Specificity = 90.91% (95% CI: 58.7-99.7)	Accuracy = 93.1% (95% CI: 81.3-98.5)

## Discussion

Our results demonstrate 11.5% true discordance between cytology and histology on core samples obtained using EUS-FNB. In all these cases, histology provided more accurate information than cytology, with the achievement of a definite diagnosis. Although the actual number of discordant cases was 9/44 (20.45%), there were four cases (mass-forming pancreatic lesions), in which cytology alone did not provide benign sub-characterization due to a lack of the architectural details needed for definitive diagnosis and hence were conclusively diagnosed with histology specimen and therefore excluded. 

In these five actual discordant results (adenocarcinoma, n=2; venous hemangioma, n=1; GIST, n=1; and follicular lymphoma, n=1), cytology failed to provide a definite diagnosis. For malignant epithelial neoplasms, mesenchymal neoplasms, neuroendocrine tumors (NET), benign epithelial neoplasms, and lymphoproliferative disorders, the cytology detection rates were 91.6%, 75%, 100%, 0%, and 50%, respectively. Given the extremely limited sample size of the latter two entities, conclusions about the utility of cytology for these cannot be drawn from the current study. For the two discordant cases of adenocarcinoma where the cytology was suspicious but not diagnostic of carcinoma, the total tumor linear dimension of the malignant glands/cell was 1.3 mm and 13.5 mm in the biopsy cores, implying that the tumor burden/volume was not a cause for this discordance. Among the remaining four discordant cases, three showed histologic features of mass-forming non-malignant inflammatory disorders (IgG4-AIP, chronic pancreatitis, and non-necrotizing epithelioid granuloma) and one was ectopic lymphatic tissue (splenule). Since these inflammatory conditions often involve extensive fibrosis and pancreatic parenchymal atrophy, cytology specimens are frequently hypocellular and non-diagnostic. Especially in the cases of AIP (n=1 in our study), cytology does not provide diagnostic architectural features, viz. obliterative phlebitis, periductal infiltrates, and storiform fibrosis, to allow for an accurate diagnosis. Only IgG4-positive plasma cells can be assessed by cytology and in isolation, they are insufficient for a definite diagnosis. Prior studies have shown varyingly low EUS-FNA/cytology sensitivity (7.9-36%) for AIP [8].

EUS-TA using FNA (to obtain aspirate) or FNB (to obtain core biopsy) is an integral technique in the assessment of GI and other non-GI malignancies/lesions. There are various studies addressing the effectiveness of FNA sampling for different GI lesions. The efficacy of EUS-FNA for the diagnosis of solid lesions of the pancreas is well-established, and a meta-analysis (33 studies, ~5000 patients) in 2012 suggested pooled sensitivity for malignant cytology to be 85% with a specificity of 98% [9]. The sensitivity increased to 91% if atypical and suspicious cytology were also included [9]. In contrast, other types of pancreatic lesions may not have such a high diagnostic yield with cytology alone, as evident from the Mayo Clinic experience, which suggested a 71.4% EUS-FNA cytology yield for the diagnosis of pancreatic NETs [10]. Similarly, FNA does not have a high yield in a few specific pancreatic lesions (AIP), lymph nodes (for lymphoma), and many non-pancreatic lesions like sub-epithelial gastric lesions (especially GIST) [11].

In contrast to the FNA needle, which captures aspirate only without much cutting, the newer FNB needles have a specialized tip with greater cutting surfaces and symmetrical needle points, which provide greater stability at the puncture site, thus increasing the yield of a visibly larger tissue sample. Thus, an FNB needle is able to better penetrate the dense tissue of GIST as well as the fibrotic tissue of AIP. The utility of an FNB needle was initially advocated for its superiority over FNA needles for core tissue acquisition of non-pancreatic lesions [11-12], but over time, it has been successfully used for pancreatic lesions, with high diagnostic accuracy with a lower number of passes [13], and has raised valid arguments if ROSE is even required when FNB is performed for solid pancreatic lesions [14]. Vergara et al. recently appraised the two approaches (FNA and FNB) and argue that FNA is effective for solid pancreatic masses, including the performance of next-generation sequencing (NGS), but FNB is more effective for the evaluation of a multitude of other GI lesions such as GIST and lymphoma (by allowing immunohistochemical studies). Moreover, when applied on the same lesion using different needles, FNB sampling provided qualitative information not reported on FNA, such as degree of differentiation in malignancy, metastatic origin, and rate of proliferation in NETs [15]. In addition, histologic tissue obtained with core-biopsies (FNB) is important for the evaluation of molecular markers and genomic profiling. Molecular profiling or mutational assays of tumor specimens could provide targeted therapies in patients with poor prognoses like pancreatic ductal adenocarcinoma (PDAC) [16]. Even for the evaluation of PCLs, FNB may capture the cyst wall, rather than simply aspirating the contents and epithelium of the GI tract. A meta-analysis comparing EUS-FNA and EUS-FNB demonstrates comparable efficacy for pancreatic masses, but FNB had higher diagnostic accuracy with a smaller number of passes. For these multiple reasons, FNB to obtain core biopsies is being increasingly preferred in clinical settings.

However, in centers without ROSE availability, it is still debatable how to best process the core sample obtained using FNB needles, whether by cytology (CytoLyt) or pathology (10% formalin). Two recent studies in April 2020 have looked into a portion of this question to assess the best cytology preparation [17-18]. While Chun et al. showed the non-inferiority of LBC to CS, with significantly less unsatisfactory specimens and less bloody background in LBC specimens, Zhou et al. revealed that LBC was more accurate and sensitive than CS alone. While these studies clarify the optimal cytology technique, interestingly, in both studies, additional core samples were obtained to improve the overall diagnostic yield. In contrast, our study is unique, as we sent core samples obtained using an FNB needle for cytology (including the creation of CS before placing the material into the CytoLyt for LBC), and an equal amount of specimen was placed in formalin (for routine histology), to compare their diagnostic yields.

It is well understood that FNA provides intact cells, FNB includes stroma and matrix, and in addition, a CytoLyt preparation preserves nucleic acids, whereas formalin fixation results in cross-linking and fragmented cells. Since a more enriched tumor cell population without intervening stroma and matrix is preferred for the performance of next-generation sequencing (NGS), cytology (CytoLyt preparation) has been traditionally considered superior, as it better preserves nucleic acids as compared to formalin. However, this myth has been recently challenged by Larson et al. who demonstrated no significant difference in the adequacy for NGS of EUS-FNB and EUS-FNA samples for pancreatic tumors [19-20]. In fact, Elhanafi et al. favor FNB, as it provided a sufficient sample for genomic testing in a higher proportion of patients (90.9% vs. 66.9% with FNA) [20]. Recently, an NGS mutational analysis comparison between cyst fluid and neoplastic surgical tissue showed high concordance, hence suggesting that if FNB is done well, it should be able to provide adequate tissue for NGS [21]. These data have taken away from cytology the distinct advantage that it traditionally carried, hence leaving us with the question if cytology should even be sent at all.

A previous Korean study on 118 suspected PDAC patients who underwent EUS-FNA reported no significant difference between cytology and histology in terms of sensitivity (87.9% vs. 81.9%; p=0.190) or accuracy. The authors concluded that cytology was more sensitive for lesions less than three centimeters (86.7% vs. 68.9%, p=0.033) [22]. In contrast, our study uses an FNB needle to obtain core tissue samples, rather than an FNA needle and analyzes non-pancreatic lesions, which are traditionally known to be more difficult to diagnose than PDAC. Our results suggest a higher diagnostic yield of histology than cytology (11.5% discordance), as described above.

There are several strengths of our study, including a uniform protocol throughout the study period, performance of EUS-FNB by a single, high-volume therapeutic endoscopist, consistent use of needle type and gauge (22-gauge FNB), and the use of the same FNB needle to place equal passes/quantity of core specimen in CytoLyt (for cytology) and formalin (for histology). Also, the procedure team (nurse and GI technician) remained consistent for the majority of the procedures, as is the institutional practice of having assigned teams to most therapeutic endoscopists, thus minimizing the chances of any inconsistent technique of sample handling. Conversely, the obvious limitations of our study include a single-center experience, which was recorded in a prospective fashion but analyzed retrospectively, and not being randomized data. Also, there is a lack of cost-analysis between cytology and histology, which is a potential area of further investigation. Being a large tertiary academic medical center, the initial cytology and histology were interpreted by multiple cytopathologists and GI pathologists, which could be argued as a limitation, but we have mitigated that factor by having a senior GI pathologist with additional cytology training retrospectively review all discordant cases.

## Conclusions

In conclusion, our study indicates 11.5% true discordance between histology and cytology, when core samples obtained from pancreatic and non-pancreatic lesions using a EUS-FNB needle are processed in formalin and CytoLyt. In all discordant cases, histology successfully provided accurate diagnosis when cytology was negative or inconclusive. Our data supports sending FNB core samples for histology (formalin) rather than for cytology, as it provides an accurate diagnosis and additionally allows the performance of NGS (just like cytology) as well as specific RNA sequencing/gene profiling of tumors. Furthermore, when a benign non-neoplastic entity is clinically suspected, the patients should undergo FNB with histology that allows architectural characterization better than cytology. Future multicentric prospective randomized studies are needed to ascertain the best practice to make the diagnostic process more efficient, accurate, universal, and cost-effective.
